# A Rapidly Deployable Test Suite for Respiratory Protective Devices in the COVID-19 Pandemic

**DOI:** 10.1177/1535676020947284

**Published:** 2020-09-01

**Authors:** Thijs Blad, Joep Nijssen, Freek Broeren, Bob Boogaard, Stefan Lampaert, Stefan van den Toorn, John van den Dobbelsteen

**Affiliations:** ^1^ Department of Precision and Microsystems Engineering, Delft University of Technology, Delft, The Netherlands; ^2^ Department of Virology, Erasmus MC, Rotterdam, The Netherlands

**Keywords:** COVID-19, protective face masks, particle penetration, breathing resistance, carbon dioxide concentration

## Abstract

**Introduction:**

The current COVID-19 pandemic has caused large shortages in personal protective equipment, leading to hospitals buying their supplies from alternative suppliers or even reusing single-use items. Equipment from these alternative sources first needs to be tested to ensure that they properly protect the clinicians that depend on them. This work demonstrates a test suite for protective face masks that can be realized rapidly and cost effectively, using mainly off-the-shelf as well as 3D printing components.

**Materials and Methods:**

The proposed test suite was designed and evaluated in order to assess its safety and proper functioning according to the criteria that are stated in the European standard norm EN149:2001+A1 7. These include a breathing resistance test, a CO_2_ build-up test, and a penetration test. Measurements were performed for a variety of commercially available protective face masks for validation.

**Results:**

The results obtained with the rapidly deployable test suite agree with conventional test methods, demonstrating that this setup can be used to assess the filtering properties of protective masks when conventional equipment is not available.

**Discussion:**

The presented test suite can serve as a starting point for the rapid deployment of more testing facilities for respiratory protective equipment. This could greatly increase the testing capacity and ultimately improve the safety of healthcare workers battling the COVID-19 pandemic.

## Introduction

The COVID-19 pandemic places high demands on the supply chains of personal protective equipment, causing a shortage of certified respiratory protective devices.^1-3^ As a result, health care organizations are resorting to alternative suppliers and initiatives to produce face masks from readily available materials.^4^ For a great deal of these uncertified masks, it is unsure whether they meet the relevant safety standards. Moreover, this shortage facilitates an illegal market for forging safety certifications that greatly jeopardizes the safety of health care personnel.^5,6^ Currently, there is insufficient testing equipment available in several parts of Europe to assess the quality of respiratory protective devices in an acceptable way. This results in major delays in testing of equipment that is needed immediately.

Worldwide, there are several standards used to determine the safety of protection masks. In Europe, the European Committee of Standardization has created several guidelines for the validation of the performance of respiratory protective devices, which are stated in the European standard norm EN149:2001+A1.^7^ According to this norm, several aspects of the functioning of the respiratory device need to be measured to assess its safety and proper functioning. Four have been identified as particularly crucial because they determine important functional safety aspects. These are the following 4 aspects:

- **Total inward leakage:** This assesses how well particles are filtered by the complete mask. Leakage from the sides of the mask due to imperfect sealing is included.
- **Penetration of filter material:** This assesses how well particles are filtered by the filter material itself.
- **Carbon dioxide content of the inhalation air:** When in use, the carbon dioxide exhaled by the user might build up between the user and the mask. This test assesses whether the concentration of carbon dioxide behind the mask does not exceed safe values.
- **Breathing resistance:** Due to the filtering properties of the material, it will add extra resistance to the inhaled air flow. This test assesses whether breathing through the mask is not excessively strenuous.

Testing these characteristics to conventional safety specifications could significantly increase trust of health care workers in obtained equipment. These masks, however, are usually tested with specialized equipment that also has to be modified depending on the type of mask.

In this article, we present a rapidly deployable test suite for all 4 aforementioned aspects of a face mask. The test suite consists mainly of 3D-printed or off-the-shelf components and centers around 3 main pieces of equipment, which are shared between the individual setups: a particle counter, a dummy head, and a flow measurement and regulation setup. By sharing equipment between different setups, the costs and construction time for the test suite are minimized. This aligns with the objective of the test suite, which is to have a fast, repeatable, and reliable method for health institutions to test their purchases and protective supplies. The total development time, including revisions and production, of this test suite was 3 weeks.

We will first describe the test setups and present our measurement protocol for each of the setups. Then, we will present the first results obtained using this test suite on a range of commercially available face masks. Finally, these results will be discussed.

## Methods

The use of interchangeable components is crucial to obtain a rapidly deployable setup, which lies at the core of the objective of this work. In the test suite, there are 3 pieces of specialized equipment:

- Fit tester: We used a Portacount pro+ 8083 used for standard fit testing.^8^
- Breathing simulator: We used a Respironics CA 3200, but this machine could easily be replaced by any device capable of creating a flow with alternating direction.
- Particle generator: A Topas ATM226 atomizer has been used to create particles necessary for the material penetration test.

The fit tester is used both for its original purpose, testing the total inward leakage of a mask when worn by a person, and as a particle counter in the material penetration test. The breathing simulator is only used in the carbon dioxide retention test, where a realistic back-and-forth flow is necessary. Besides these existing components, 3 other components are shared between testing setups with only minor modifications:

- dummy head: a 3D-printed realistic human head^9^, which is coated in silicone rubber to simulate skin;
- controlled fan: used to imitate volumetric flows that occur during breathing;
- flow sensor: a flow sensor based on a venturi nozzle.

All these components have been constructed using 3D printing and only require commonly available electrical components. Using these pieces of equipment, along with several connectors, the complete test suite can be constructed.

### Breathing Resistance

**Figure 1.**
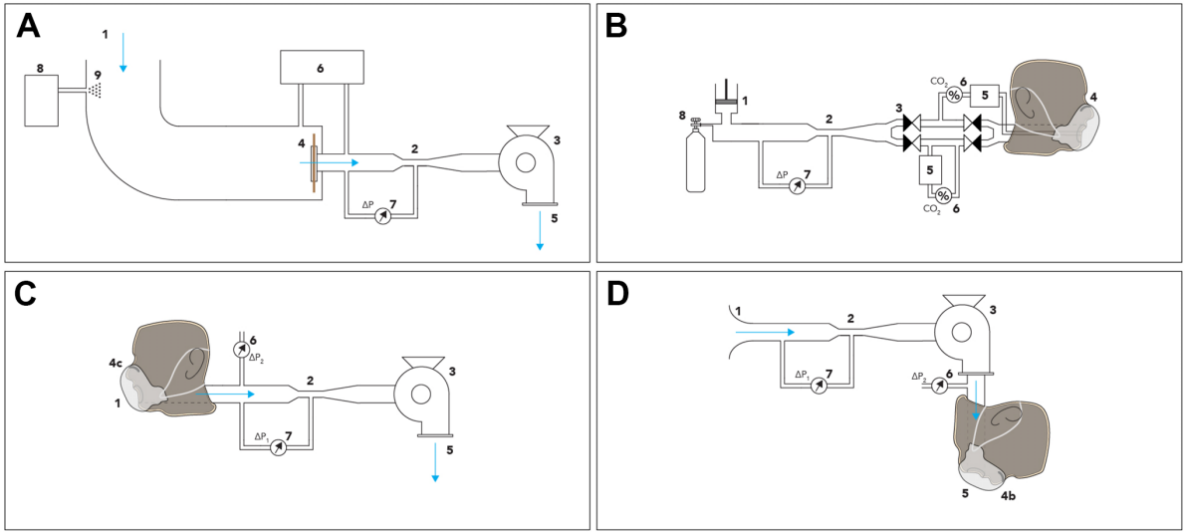
Test suite for validation of performance of respiratory protective devices according to EN 149:2001+A1. (a) Setup of penetration of filter material with (1) incoming filtered air, (2) restriction, (3) pump, (4) clamped mask, (5) pump output flow, (6) particle counter, (7) flow sensor, (8) particle generator, (9) input location particles. (b) Carbon dioxide content of the inhalation air with (1) breathing simulator, (2) venturi nozzle, (3) check valves, (4) dummy head, (5) reservoir, (6) CO2 sensor, (7) differential pressure sensor, (8) CO2 supply, (9) fan. (c, d) Breathing resistance, (1) air inlet, (2) venturi nozzle, (3) radial fan, (4b/c) dummy head with protective mask, (5) air outlet, (6, 7) differential pressure sensors.

The 2 measurements performed to characterize the breathing resistance of the face mask are depicted schematically in Figure 1c and 1d and make use of the dummy head, controlled fan, and flow sensor. The breathing resistance over the whole mask is measured by attaching the mask on a dummy head where air is pumped through the mouth to simulate breathing. Using this method, both inhalation and exhalation resistance are measured. In the exhalation setup, the dummy head in Figure 1c, component 4b, is mounted on the air outlet of the system, where it replaces the membrane. The pressure drop is measured by a differential pressure sensor (6) with an input in the ambient air and the other input directly before the dummy head. In the inhalation setup, the dummy head seen in Figure 1c, component 4c, is mounted on the air inlet of the system, and the differential pressure sensor (6) is moved such that it has one input directly after the dummy head. The other input remains in the ambient air. In accordance with the European standard, the maximum allowable pressure difference for different load cases can be seen in Table 1.

**Table 1.** Maximum Allowable Pressure Differences (Pa) for the Different Protection Levels of Masks in Accordance with EN-149.

Class	Inhale 30 L/min	Inhale 95 L/min	Exhale 160 L/min
FFP1	60	210	300
FFP2	70	240	300
FFP3	100	300	300

### Penetration of Filter Material

For the penetration of filter material test, a number of components and functionalities have been changed with respect to the conventions of the norm. The main difference is the use of a particle counter instead of a flame photometer used to measure sodium concentrations, which is an approach that has been seen before in literature.^10^ The setup proposed here is therefore an approximation of the norm because the mechanism of measurement is different. Instead of measuring solely the sodium concentration, the setup looks at the relative concentration of the total particles before and after filtration that are similar to those presented in the norm. The comparable method is therefore used to determine the penetration of particles through the mask.^7^ The average velocity is based on the effective surface of protective masks used in this work and the required volumetric flow stated in the norm. Because in the proposed setup only particles can be counted, a comparison needs to be made between total particle mass flow and flow rate. If this ratio is similar in the proposed setup, it is assumed a comparable load case in terms of concentration is given. In the standard, it is required to measure the entire mask. The method we propose in this work is to instead only measure a part of the mask. This makes the comparison focused on the material while keeping the size of boundary equipment low. It is done by having a specific clamp for the protective masks within a tube in which the aerosol concentration is provided, as can be seen in Figure 1a. A particle generator is used to create the required NaCl concentration, seen in Figure 1a.

This setup is an extension of the previously presented resistance measurement principle, similar in mask placement as seen in Figure 1d. The atomizer present is required to create particles between 0.06 µm and 0.1 µm. By measuring the particle count before and after the mask, the P value can be determined. This can then be checked in accordance to the European standard. Because the measurement principle is different, it is approximate to the same allowable standard percentages. These percentages, in accordance with the European standard, can be found in Table 2.

**Table 2.** Allowable Percentages of Leakage in Accordance with EN-149.

Class	Maximum Leakage for a NaCl Test at 95 L/min
FFP1	20%
FFP2	6%
FFP3	1%

### Carbon Dioxide Content in Inhaled Air

This setup is a simplified alternative of the setup specified in section 8.7 of the EN-149. To measure the carbon dioxide buildup behind the mask, the system depicted schematically in Figure 1b is proposed. During this test, the breathing simulator (1) induces exhalation and inhalation cycles of 2 L air at 25 times per minute. These flows are forced through a venturi nozzle (2), a system of check valves (3), and through the face mask attached to a dummy head (4). Part of the exhalation and inhalation flows are directed through a reservoir (5) and a CO2 sensor (6) to measure their CO2 content. A differential pressure sensor (7) is connected to the venturi nozzle to measure the flow during inhalation and exhalation. A supply of CO2 (8) is used to maintain the CO2 content during the exhalation at 5%, and a fan (9) is used to provide a slight air flow around the dummy head, which prevents the buildup of a cloud of CO2 in front of the mask. All masks function according to the standard when the carbon dioxide content of the inhalation air does not exceed an average of 1.0% by volume.

### Realization of Setups

**Figure 2.**
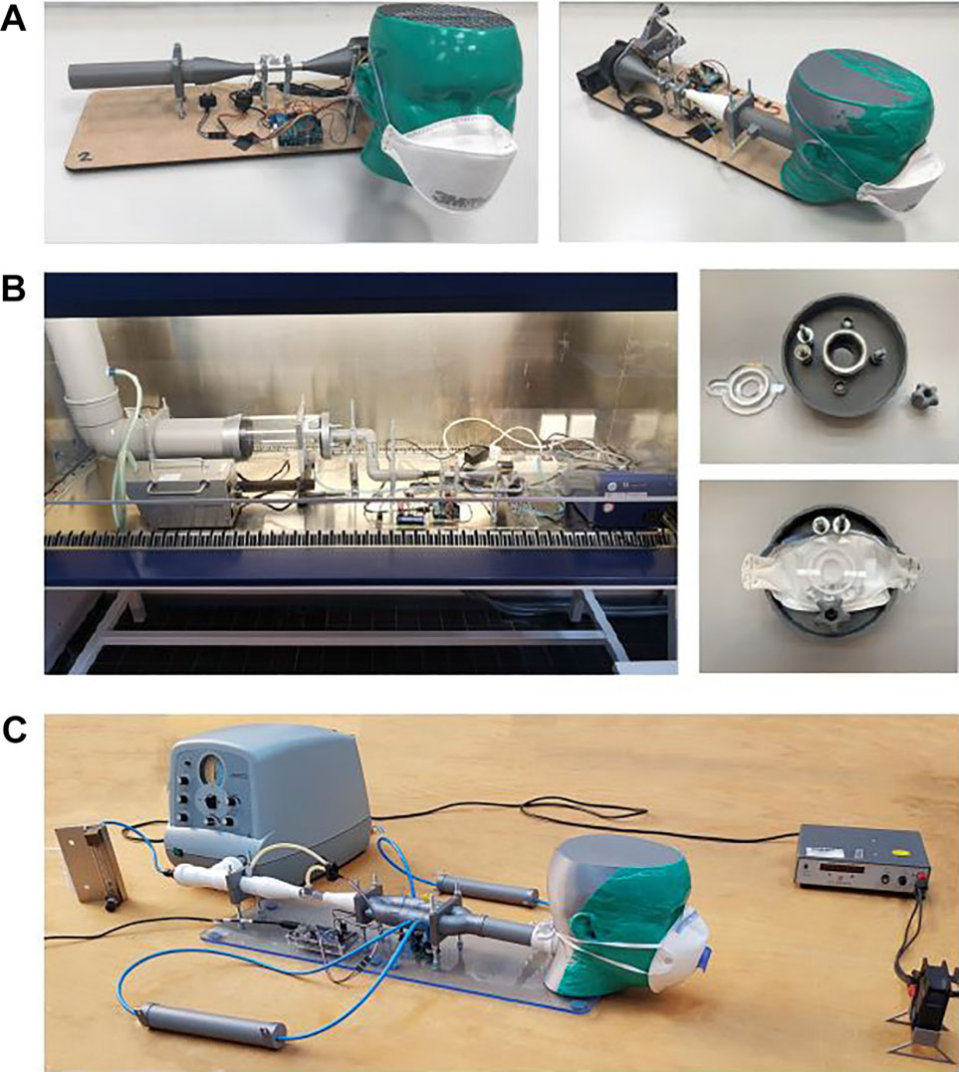
Realized setups for the test suite for validation of performance of respiratory protective devices according to EN 149:2001+A1. (a) Breathing resistance inhalation setup and exhalation setup. (b) Penetration of filter material. (c) carbon dioxide content of the inhalation air.

The designs described previously and shown in Figure 1 have been constructed and were used to test a range of commercially available face masks. Photos of the realized setups are shown in Figure 2.

#### Breathing resistance

An overview of the measurement setups that are used in the breathing resistance tests is shown in Figure 2c. It consists of a pump, 2 differential pressure sensors, an Arduino Uno, and tubing. The pump (Sanyo Denki 9BMB24P2H01) is a radial fan that can deliver a static pressure of 490 Pa. The pressure sensors (Sensirion SDP816-500PA) are differential pressure sensors that can measure pressure differences up to 500 Pa. The system is controlled using an Arduino Uno to set the fan speed and collect data from the sensors.

The narrow section of the venturi nozzle was made of an aluminum tube with an inner diameter of 12 mm. The remaining parts, such as brackets, nozzles, and diffusers, were 3D printed in PLA. The designs of these parts can be found in the Supplemental Material. In the membrane test, a piece of filter material from the mask is clamped in the membrane mount by a piece of acrylic and tightened by 2 bolts. The membrane mount forces the air through an opening with a diameter of 40 mm. Measuring the air through a fixed area allows for the testing of the resistance of the membrane material independent from mask shape, size, and fit. A program was written for the Arduino that sweeps the fan speed from standstill to maximum and captures the data from the pressure sensors, which can be found on https://projectmask.nl/. This program was used for all of the tests. For the inhalation and exhalation tests, the whole mask is mounted on a dummy head according to fitting instructions of the manufacturer. Special care is taken to tighten the metal nosepiece to ensure a good fit. To approximate the head of a real test subject, a dummy head was 3D printed and coated with silicone rubber. A tube of d_i_ = 32 mm, d_o_ = 40 mm is fitted from the mouth to the back of the head to connect it to the setup. The dummy head used in this study is from Bergman et al,^9^ to provide an accurate representation of an average head. The shape of the dummy head in that study was based on the work of Zhuang and Bradtmiller,^11^ who developed an anthropometric database of heads and faces of US civilians utilizing face masks during their work. From 1013 members, the faces were digitized using 3D scans. In this way, industry was provided a more accurate face mask, nicely fitting on most faces. A silicone rubber is used to mimic the properties of the skin, consisting of a 2-component molding rubber with a short curing time.

#### Penetration of filter material

The embodiment of the setup can be seen in Figure 2a. The previously presented breathing resistance has been integrated to provide the controllable volumetric flow. The output of that subsystem remains free, similar to the inhalation test. Additionally, the setup makes use of a set of tubes used to provide dry air including particles toward the mask. The mask is clamped with a restrictor plate used to control the input velocity of the mask. The mask is clamped onto the setup as seen in Figure 2a/b and sealed to prevent leakage of the environment after the mask. In a similar fashion to the breathing resistance test, all Supplemental Material files are provided. The setup is placed underneath a HEPA-filtered fume hood to provide a supply of clean dry air with a constant input flow. The humidity is measured inside the tube using a portable humidity meter in front of the mask. The particle counter used in this setup is a TSI portacount pro+ 8083, which is part of a standard test setup for the fit tests. The atomizer used is the previously mentioned Topas ATM226 series atomizer aerosol generator. This has a maximum mass flow of 2.5 g/h, able to create particles with an average particle size of 0.07 (µm) with a geometric standard deviation GSD of 2 (–). The restrictor diameter to determine the velocity at the mask is 24 mm. A number of variables have influence on the results obtained by this measurement, namely, the particle concentration and suction velocity at the mask input. The setup was solely validated using the NaCl solution as particles. A specified load case is thus needed to approximate the load condition stated in the European standard.

#### Carbon dioxide content in inhaled air

An overview of the measurement setup that is used in the CO2 test is shown in Figure 2b. The setup consists of a breathing simulator, a differential pressure sensor, 2 CO2 sensors, a CO2 supply, a dummy head, an Arduino Mega, and tubing including check valves. The breathing simulator (Respironics CA 3200) is used to generate alternating cycles of inhalation and exhalation of 2 L each, 25 times per minute. The airflow fed through the CO2 sensors (GSS SprintIR-WF-20) is premixed in a reservoir with a volume of 100 ml to average the CO2 concentration. Around the dummy head, a flow of 0.5 m/s is generated by an 80-mm PC fan. The differential pressure sensor, venturi nozzle, and dummy head are as discussed in the next section. During operation, the sensor values are collected using an Arduino Mega. The remaining parts, such as brackets, tubing connections, and check valves, were 3D printed in PLA. The designs of these parts can be found in the Supplemental Material.

#### Test protocol

As presented, the objective of the test suite is to provide a fast, repeatable approach to determining the efficacy of masks. Depending on the type of mask, a different approach to testing should occur. In this work, we differentiate between the following:

- **recycled masks:** masks that are known to the buyer but have been recycled, for instance, using steam sterilization or any other recycling process being developed;
- **validated masks:** masks that are bought from a (un)known seller, claiming full functionality in accordance with their respective classification and international standards;
- **unvalidated masks:** masks that have not been tested according to existing standards.

Depending on these principle mask types, the following test procedure is proposed:

- **recycled masks:** inwards leakage test (using fit 8), followed by filter material penetration test;
- **validated masks:** inwards leakage test (using fit 8), followed by filter material penetration test;
- **unvalidated masks:** inwards leakage test (using fit 8), followed by CO2 content test, breathing resistance test, and ending with filter material penetration.

The resulting combination of test results can then be used to determine the efficacy of the protective mask in question. The specific protocol steps for the inward leakage test, filter material penetration test, breathing resistance test, and carbon dioxide test can be found online at https://projectmask.nl/.

## Results

To present how the test suite could be utilized, 6 masks of different classifications were tested. They will be used to present typical results obtained with the test suite. Of the 6 samples used, 3 are FFP2 classified, 1 is FFP3 classified, 1 is KN95 classified,^12^ and the final sample (surgical mask) has no filtering classification. The last is used as a baseline, which should further confirm its lack of filtering, as has been seen in Bowen.^13^

### Breathing Resistance

Using the load case specified in EN-149, the resulting pressure drop for all these masks can be seen in Table 4.

**Table 3.** Load Case Used with Respect to the Load Case Proposed in the EN149 Standard.

Property	Case	EN149
Particle mass flow (mg/min)	6.25	57
Volume flow (L/min)	7.5	95
Velocity (m/s)	0.25	N/A
Concentration NaCl (%)	2	2

**Table 4.** Inflow and Outflow Rates and Pressure Differences (Pa) as Results of the Breathing Resistance Tests.

Mask Model	Inhale 30 L/min	Inhale 95 L/min	Exhale 160 L/min
FFP3 Dräger piccolo	25.1	111.4	288.8 (150L/min)
FFP2 3 M 8320	21.3	85.9	227.7
FFP2 3 M 1862	21.3	90.1	216.0
FFP2 3 M aura 9320+	22.4	118.6	243.2
KN95 Purvigor	13.0	61.0	148.3
Medline surgical mask	11.5	61.9	136.3

### Penetration of Filter Material

Because this setup does not measure the entire mask but only a part, the velocity becomes of importance. Because the EN149 measures an entire mask, it does not provide a usable velocity value that can be used. Also in literature, there are varying velocities presented,^14^ although in general, they are considered low at less than 0.1 m/s. From other literature, it can be observed that the velocity during breathing varies greatly, as seen in Tang et al^15^ and Anthony and Anderson.^16^ Based on Anthony and Anderson,^16^ the load case used to verify the efficacy of the masks is defined by a velocity of 0.25 m/s, which can be considered a harsher case compared to others seen in literature. The used load case in this work, in comparison to the standard, can be seen in Table 3.

The test protocol is similar to that provided in EN149 and EN13274-7^17^, which is an accompanying standard for this test. This means the average value over a period of 30 seconds is determined while maintaining a humidity below 40% at room temperature. The presented values in Table 3 show a comparable case between the rapid deployable setup and the EN149 described standard. The particle mass flow to volumetric flow is higher, with a factor 0.83 with respect to that of the EN149 standard at 0.6. The protocol is repeated 3 times per mask. Results and the average P-factor can be found in Table 5.

**Table 5.** Resulting Leakage for Specified Load Case for 3 Test Runs, Denoted C_1_, C_2_ and C_3_, Respectively.

Mask Model	C_1_	C_2_	C_3_	Average
FFP3 Dräger piccolo	0.1%	0.11%	0.095%	P = 0.1%
FFP2 3 M 8320	0.77%	0.8. %	1.11%	P = 0.9%
FFP2 3 M 1862	0.8%	1.22%	1.05%	P = 1.02%
FFP2 3 M aura 9320+	1.33%	1.47%	1.59%	P = 1.46%
KN95 Purvigor	14.49%	19.6%	22.73%	P = 18.94%
Medline surgical mask	40%	47.62%	45.45%	P = 44.36%

^a^ The average filter percentage can be compared to the standard for each given classification of Table 2 to determine their performance. The KN95 Purvigor type mask is the only one that does not reach is specified classification carbon dioxide content in inhaled air.

The test protocol for the setup is similar to that provided in EN149. However, instead of 3 different masks, 1 mask is tested 3 times because of availability. The setup is turned on before starting the measurement. After 1 minute, the mask is put on the dummy and removed 1 minute later. This is done 3 times, after which the measurement is stopped. Table 6 shows the average CO2 inhalation concentration for different half face masks. The surgical mask was not taken into account because, due to the loose fit, it has no dead space and therefore no measurable carbon dioxide content.

**Table 6.** Resulting CO2 Concentrations for the Tested Masks.

Mask Model	CO2 Exhale Concentration	CO2 Inhale Concentration
FFP3 Dräger piccolo	5.47%	0.94%
FFP2 3 M 8320	4.74%	0.84%
FFP2 3 M 1862	5.32%	0.90%
FFP2 3 M aura 9320+	5.05%	0.48%
KN95 Purvigor	5.21%	0.42%

## Discussion

### Breathing Resistance

It can be observed from Table 4 that differences can be found between the breathing resistances of the masks, where certified masks with more filter capacity showed a greater breathing resistance. Because the breathing resistance test can be conducted very rapidly, the breathing resistance could possibly serve as a first indication for the fit and the filter properties of a mask. The measured volumetric flow was seen to be fluctuating more significantly with higher resistance masks. To solve this, a moving average filter was implemented in the program to stabilize the volumetric flow sensor.

### Penetration of Filter Material

Table 5 shows an expected general trend between FFP2 and FFP3 masks. The only tested FFP3 model simultaneously performed the best with a substantial increase in P-factor with respect to the other samples. As for the remaining masks, all performed within their required EN-149 standard, with the KN95 Purvigor being the exception. This type, although stated as being KN95, which is similar to an FFP2 classification, only performed on a low FFP1 classification level. The surgical mask, as expected, performed the worst out of the test set, which also in literature showed very low filtering capacity.^13^ The setup is repeatable, although the error between separate measurements increases with lower filtering capacity. This is to be expected because the relative error in filtering causes a larger distribution in measured filtering capacity.

It was observed that the measurement principle is not suitable for repeated tests of the same sample due to saturation of the filter material. A different FFP2 3 M 8320 was used for the initial setup tests and therefore exposed to a significant number of test runs. This caused saturation of the mask, which decreased filtering to ≥25%, meaning lower than FFP1 classification.

### Carbon Dioxide Content in Inhaled Air

Results in Table 6 show that for all tested masks, a concentration of less than 1% CO2 was measured during inhalation. As a result, the buildup of CO2 behind these masks is within the limits of EN149. The values are slightly higher than would be expected based on the dead volume added by the half face masks, which can be caused the large opening in the dummy.

### Applicability of the Test Suite

The results obtained with the rapidly deployable test suite agree with conventional test methods, demonstrating that this setup can be used to assess the filtering properties of protective masks when conventional equipment is not available. It does not, however, contain all measurements as detailed in the EN149 standard and is therefore not a suitable replacement. Furthermore, no precondition or simulated wear steps were included in these measurements.

Based on our experience developing, testing, and revising the test suite, we expect that the deployment time of a new setup, using the material available on https://projectmask.nl, can be under 1 week.

## Conclusion

In this work, a measurement setup suite was developed for running a series of tests to characterize the breathing resistance, filter material penetration, and carbon dioxide content of protective masks. The test suite approximates the EN149 standard and can therefore provide valuable validation of unknown or recycled protective face masks during the COVID-19 pandemic. The 6 samples show the expected trends between FFP2 and FFP3 masks but also showed that we need to be careful when implementing protective masks because it is possible for FFP2 comparably labeled masks to not function up to even FFP1 classification. Continuous efforts will be made to test a wide variety of masks, including recycled masks, of which the data will be made available on https://projectmask.nl/.
